# Comparison of Clinical and Hematological Parameters of Janus Kinase 2, Calreticulin or Myeloproliferative Leukemia Virus Oncogene Mutant Essential Thrombocythemia and Triple-Negative Essential Thrombocythemia

**DOI:** 10.7759/cureus.23171

**Published:** 2022-03-15

**Authors:** Jale Yıldız, Hikmettullah Batgi

**Affiliations:** 1 Department of Hematology, Yıldırım Beyazıt University, Yenimahalle Training and Research Hospital, Ankara, TUR; 2 Department of Hematology, Ankara Training and Research Hospital, Ankara, TUR

**Keywords:** mpl, polycythemia vera, jak2, essential thrombocythemia, chronic myeloproliferative neoplasms

## Abstract

Introduction

Essential thrombocythemia (ET) is one of the chronic myeloproliferative neoplasms. While Janus kinase 2 (JAK2) V617F mutation is defined in more than half of the patients with ET, calreticulin (CALR) or myeloproliferative leukemia virus oncogene (MPL) mutations are encountered more rarely. The discovery of the JAK2 V617F mutation in 2005, followed by the recognition of MPL and CALR mutations, brought up the idea of subdividing ET according to the mutation status. Our aim in this study is to investigate whether genetic mutations detected in patients diagnosed with ET cause a different clinical phenotype compared to triple-negative ET.

Methods

This retrospective study was conducted by evaluating the patients who were followed up with the diagnosis of ET in the hematology clinic of two tertiary centers in Turkey between 2009 and 2021. Patients with negative JAK2, CALR, and MPL mutations and meeting the diagnostic criteria for ET were defined as triple-negative ET. The patients were divided into two groups as triple-negative ET and mutation-positive ET according to the presence of a mutation. It was investigated whether there was a difference between these two groups in terms of demographic, laboratory, and clinical characteristics.

Results

A total of 109 patients were included in the study. The mean age of these patients was 54 (18-91) years and 85 (78%) patients were females. A total of 48 patients (44.0%) had JAK2 mutation, six (5.5%) had CALR mutation, and one (0.9%) had MPL mutation. It was observed that there was a significant difference between the two groups in terms of gender, mean age, and hemoglobin value. While 87% of patients with triple-negative ET were females, this rate was 69% in patients with mutation-positive ET (p = 0.036). The mean age was 41.8 years in triple-negative ET and 67.1 years in patients with mutation-positive ET (p = 0.0001). While the mean hemoglobin value was 12.9 g/dl in patients with triple-negative ET, it was 14.4 g/dl in patients with mutation-positive ET (p = 0.0001).

Conclusion

It has been observed that ET with JAK2, CALR, or MPL mutations may have different phenotypic features compared to triple-negative ET, resulting in a clinical condition consisting of older patients with a higher erythrocyte count.

## Introduction

Chronic myeloproliferative neoplasms are hematopoietic stem cell diseases that present with clonal proliferation in the erythroid, myeloid, and megakaryocytic lineages [1]. According to the World Health Organization (WHO) 2016 classification, three diseases associated with the Janus kinase 2 (JAK2)/calreticulin (CALR)/myeloproliferative leukemia virus oncogene (MPL) mutation (polycythemia vera, essential thrombocythemia (ET), and primary myelofibrosis) and four clinicopathological diseases (chronic myeloid leukemia, chronic neutrophilic leukemia, chronic eosinophilic leukemia, and not otherwise specified-unclassifiable) are included in chronic myeloproliferative neoplasms [1,2].

ET is one of the chronic myeloproliferative neoplasms, characterized by reduced quality of life and thrombohemorrhagic complications, as well as a risk of transformation into myelofibrosis and acute leukemia [3]. There are no recently published data on the true incidence of ET. Its annual incidence in the United States is estimated at 2.5 cases per 100,000. In Europe, this rate is thought to vary between 0.2 and 2.5 per 100,000 [4,5]. According to retrospective evaluations, it was observed that approximately 5% of patients with thrombocytosis were diagnosed with ET [6]. It is estimated that it is observed about two times more frequently in women than in men [7].

According to the definition made by the WHO in 2016, an increase in mature megakaryocytes in the bone marrow with high platelet count, the absence of causes that will cause reactive thrombocytosis, and the absence of other myeloproliferative neoplasms are among the diagnostic criteria for ET. Apart from these, mutation of JAK2 V617F, CALR in exon 9, or MPL in exon 10 have also been defined by the WHO as major diagnostic criteria [1,2,8].

While JAK2 mutations are defined in more than half of the patients with ET, CALR and MPL mutations are encountered more rarely [1-3,9,10]. The discovery of the JAK2 V617F mutation in 2005, followed by the recognition of MPL and CALR mutations, brought up the idea of subdividing ET according to the mutation status [1-3]. Retrospective reports indicating that triple-negative ET with negative JAK-2, MPL, and CALR mutations show different clinical features compared to patients with mutation-positive ET has increased the interest in this subject [11-16].

Our aim in this study is to investigate whether genetic mutations detected in patients diagnosed with ET cause a different clinical phenotype compared to triple-negative ET. If a clinical difference is detected between these groups as a result of the study, it will be thought that there may be differences in the follow-up of patients diagnosed with ET according to the presence of genetic mutations.

## Materials and methods

This retrospective study was conducted by evaluating the patients who were followed up with the diagnosis of ET in the hematology clinic of two tertiary centers in Turkey between 2009 and 2021. Before the study, approval was obtained from Ankara Yıldırım Beyazıt University Ethics Committee (approval number: E-2022-11).

According to the WHO 2016 criteria, platelet count ≥ 450 × 109/L, proliferation in megakaryocyte series in bone marrow biopsy, exclusion of other myeloid neoplasm diagnoses, and presence of JAK2, CALR, or MPL mutation were accepted as major criteria. The absence of a cause for reactive thrombocytosis and the positivity of a clonal marker were also defined as minor criteria. Presence of all four major criteria or one minor criterion in addition to three major criteria were considered necessary for the diagnosis of ET [2].

Patients who were diagnosed with ET according to the WHO criteria, older than 18 years, male and female, and who had access to clinical and laboratory information were included in the study. Patients who did not meet the WHO 2016 ET diagnostic criteria, were younger than 18 years, had missing clinical information in the patient file, had unknown genetic mutation status, and had positive BCR/ABL mutations were excluded from the study.

Demographic characteristics (age and gender), ET characteristics (history of diagnosis of essential thrombocytosis and treatments received), comorbid diseases, and ET-related complications (bleeding and thrombosis) of the patients included in the study were investigated through manual patient files and an electronic patient registry system. Apart from these, hematological parameters (hemoglobin, platelet, and white blood cell count), mutation status (JAK2, CALR, and MPL), and presence of radiologically proven splenomegaly were also recorded.

Patients with negative JAK2, CALR, and MPL mutations and meeting the diagnostic criteria for ET were defined as triple-negative ET. The patients were divided into two groups as triple-negative ET and mutation-positive ET, according to the presence of a mutation. It was investigated whether there was a difference between these two groups in terms of demographic, laboratory, and clinical characteristics.

Obtained data were analyzed by IBM Statistical Package for the Social Sciences (SPSS®) v.21 (IBM Corp., Armonk, NY). Demographic data are summarized with descriptive statistics. Numerical variables are presented as mean (minimum and maximum). Mann-Whitney U test was used for numerical variables and chi-square test was used for categorical variables to compare groups. Statistically, the p-value of <0.05 was accepted as significant.

## Results

A total of 153 patients with a diagnosis of ET were evaluated for eligibility for the study. Of these, 27 were excluded because they did not meet the WHO 2016 ET diagnostic criteria, 10 did not have JAK2, CALR, or MPL testing, and the other seven patients were excluded because their medical records were incomplete.

A total of 109 patients were included in the study. The mean age of these patients was 54 (18-91) years and 85 (78%) patients were females. Mean platelet was 710 × 109/L (461-1,671 × 109/L), mean leukocytes were 10.3 × 109/L (4.7-18.3 × 109/L), and mean hemoglobin level was 13.7 g/dl (10.8-18.0). A total of 48 patients (44.0%) had JAK2 mutation, six (5.5%) had CALR mutation, and one (0.9%) had MPL mutation. The number of patients who were defined as triple-negative ET and did not have a mutation was 54 (49.5%). When ET-related complications and clinical events were examined, it was found that 22 (20.2%) patients had thromboembolism and 15 (13.8%) patients had splenomegaly. Comorbidities were present in 50.5% (n = 55) of the patients. The most common comorbid disease was hypertension (n = 34, 31.2%). Due to ET, 46.8% (n = 51) of the patients were receiving acetylsalicylic acid and hydroxyurea, and 44% (n = 48) were receiving acetylsalicylic acid alone. Patient characteristics are shown in Table 1.

**Table 1:** Characteristics of the patients. JAK2, Janus kinase 2; CALR, calreticulin; MPL, myeloproliferative leukemia virus oncogene.

Variables	(n = 109)	%
Age (years), mean (min-max)	54 (18-91)	
Gender		
Female	85	78.0
Male	24	22.0
Genetic mutation		
JAK2	48	44.0
CALR	6	5.5
MPL	1	0.9
Lack of demonstrable mutations	54	49.5
Comorbidities	55	50.5
Thrombosis	22	20.2
Splenomegaly	15	13.8
Laboratory findings, mean (min-max)		
Platelets	710 × 10^9^/L	461-1671 × 109/L
Hemoglobin	13.7 gr/dl	10.8-18.0 gr/dl
White blood cells	10.3 × 10^9^/L	4.7-18.3 × 10^9^/L

When the patients were divided into two groups as triple-negative ET and mutation-positive ET and their demographic and clinical characteristics were compared, it was observed that there was a significant difference between the two groups in terms of gender, mean age, and hemoglobin (Table 2). While 87% of patients with triple-negative ET were females, this rate was 69% in patients with mutation-positive ET (p = 0.036). The mean age was 41.8 years in triple-negative ET and 67.1 years in patients with mutation-positive ET (p = 0.0001). While the mean hemoglobin value was 12.9 g/dl in patients with triple-negative ET, it was 14.4 g/dl in patients with mutation-positive ET (p = 0.0001). There was no difference between the two groups in terms of other clinical findings (Table 2).

**Table 2:** Comparing clinic and laboratory findings of triple-negative and mutation-positive essential thrombocythemia (ET).

Variable	Triple negative ET (n = 54)	Mutation positive ET (n = 55)	P-value
Age (years), mean	41.8 (21-67)	67.1 (25-84)	0.0001
Female	47 (87.0%)	38 (69.1%)	0.036
Comorbidity	26 (48.1%)	29 (52.7%)	0.703
Thrombosis	11 (20.4%)	11 (20.0%)	0.132
Splenomegaly	4 (7.4%)	11 (20.0%)	0.09
Laboratory findings, mean			
Platelets	702 x 10^9^/L	718 x 10^9^/L	0.171
Hemoglobin	12.9 gr/dl	14.4 gr/dl	0.0001
White blood cells	9.8 x 10^9^/L	10.8 x 10^9^/L	0.126

## Discussion

In this study, in which we investigated the clinical and hematological differences between mutation-positive ET and triple-negative ET, it was observed that patients with triple-negative ET had a significant female predominance, and patients with JAK2, CALR, or MPL mutations were diagnosed at an older age. In addition, it was observed that hemoglobin level was higher in patients with mutations compared to those with triple-negative ET. The result we obtained suggests that patients with JAK2, CALR, or MPL mutation-positive ET may have polycythemia vera.

ET is one of the chronic myeloproliferative neoplasms characterized by high platelet count, negative Philadelphia chromosome, thrombotic events, and bleeding risk [9]. The JAK2 mutation, which was identified in the early 2000s, was followed by the discovery of CALR and MPL mutations [1]. It has been reported that JAK2 mutation is positive in approximately half of the patients with ET, and CALR and MPL mutations are detected more rarely (<10% and <5%, respectively) [1,10]. In our study, JAK2, CALR, and MPL mutations were found to be positive in approximately 45%, 5%, and 1% of patients, respectively. These rates appear to be slightly lower than previous studies in the west [12,13,15]. However, in a study conducted in Asia that included approximately 100 ET cases, the JAK2 mutation was found to be positive in 34% of the patients, which was lower than that in our patient group [17]. The fact that the studies we mentioned were conducted in different geographies and included patients from different ethnic groups causes the difference in the frequency of JAK2 mutations.

According to the most recent regulation of the WHO, JAK2, CALR, and MPL mutations are each among the diagnostic criteria for ET [2]. There is another subtype defined as triple-negative ET in which all three mutations are negative [11]. But do these mutations cause different phenotypes of ET? Do patients with unmutated triple-negative ET have a different clinic? These questions have been the main subject of many studies for about 15 years [11-16].

The first remarkable results of our study were that patients with mutation-positive ET were older than those with triple-negative ET, and female predominance was more pronounced in terms of gender distribution. Our results were generally consistent with previous studies. One of the first comprehensive studies investigating the clinical implications of mutational status in patients with ET was a series of approximately 800 patients published by the University of Cambridge [13]. In this study, it was reported that patients with ET with JAK2 mutation were significantly older than those with negative JAK2 mutation. Similarly, in a study involving approximately 100 patients with ET in Asian patients, it was mentioned that those with JAK2 mutants were at an older age and mutation positivity was more common in women [17]. In the experiences in Italy, it has been reported that patients with JAK2 mutations are at an older age than those with mutation negatives [18,19]. Unlike other studies, a more recent study in which the CALR mutation was also examined was conducted in Belgium. In this study, which included approximately 150 patients with ET, it was observed that the JAK2 mutant group was older than CALR mutant and triple-negative patients [20].

One of the significant results obtained in our study was that the hemoglobin level was higher in patients with JAK2, CALR, or MPL mutations compared to patients with triple-negative ET. Many studies have been conducted on the effect of JAK2 mutation on hematological parameters in patients with ET. In the study of Campbell et al., which has the largest number of patients, it was reported that the hemoglobin, leukocyte, and neutrophil counts were higher in the JAK2 mutant group, and the platelet count was lower [13]. The fact that the CALR and MPL mutational status of the patients was unknown was one of the limitations of this study. In another study with a similar design but with a more limited number of patients, it was observed that patients with JAK2 mutation-positive ET had higher hemoglobin levels than those with negative JAK2 mutations [12]. In a study conducted in China, but with a limited number of patients, it was shown that the hemoglobin level is higher in patients with ET with positive JAK2 mutation. As a result of this study, it was suggested that JAK2-positive ET might present a clinical similarity to polycythemia vera [14]. In another study conducted in our country with a similar number of patients, the hemoglobin level was found to be significantly higher in the JAK2 mutation-positive group [15]. In the results obtained by Wong et al. from Asian patients, while the leukocyte count was higher in the mutant group, no correlation could be established between hemoglobin and thrombocyte levels and the mutation status [17]. In a very large series including patients with polycythemia vera and ET conducted in Italy, unlike other studies, JAK2 mutants were divided into heterozygous and homozygous mutants and their clinical implications were investigated. In this study, 57% of 639 ET patients had heterozygous JAK2 mutations, 2% had homozygous JAK2 mutations, and 40% were wild type. It was observed that those with homozygous JAK2 mutation had higher hemoglobin and leukocyte counts [18]. In a large Italian series including 260 ET patients, it was observed that those with JAK2 mutants exhibited a clinic with high hemoglobin and leukocyte counts and a lower platelet count [19]. Similar results were obtained in a study by Al Assaf et al., which included patients with CALR mutations [20]. The fact that patients with ET with the mutation have higher hemoglobin levels seems to be the common result of almost all studies, including ours. However, the effect of JAK2 and other mutations on platelet and leukocyte levels in patients with ET still seems controversial. In our study, there was no difference in leukocyte and platelet counts between patients with mutations and triple-negative patients.

Splenomegaly and thrombotic events are common clinical conditions in patients with ET. The relationship of JAK2, CALR, and MPL mutations with thrombosis and splenomegaly is not clear. In a study conducted in England investigating the clinical reflections of JAK2 mutation in patients with ET, thrombotic events were found to be significantly higher in the JAK2 mutant group [12]. In a study by Vannucchi et al. with a large number of patients, it was reported that splenomegaly was more common in the JAK2 mutant group [18]. In a study by Antonioli et al., also from Italy, involving more than 250 patients, no difference was found between positive and negative groups for JAK2 mutation in terms of splenomegaly and thrombotic events [19]. As can be seen, there are conflicting data regarding the association of JAK2, CALR, and MPL mutations with splenomegaly and thrombosis in patients with ET. In our study, although numerically more splenomegaly was observed in patients with JAK2, CALR, or MPL mutations, this difference was not statistically significant. There was no difference between the groups in terms of thrombosis.

The most important limitations of our study were its retrospective nature and a relatively small number of patients. The low number of CALR and MPL mutation-positive patients prevented the patients from being divided into subgroups according to the mutation type. However, when compared to studies on the same subject in the literature, our study is one of the rare examples that includes results for all three mutation types in more than 100 patients and investigates the effect of this on the phenotype.

## Conclusions

It has been observed that ET with JAK2, CALR, or MPL mutations may have different phenotypic features compared to triple-negative ET, resulting in a clinical condition consisting of older patients with a higher erythrocyte count. In future studies involving a larger number of patients, CALR and MPL mutation-positive patients can also be considered in separate groups, and the effects of genetic subgroups on the ET phenotype can be evaluated.
